# Regulatory interactions for iron homeostasis in Aspergillus fumigatus inferred by a Systems Biology approach

**DOI:** 10.1186/1752-0509-6-6

**Published:** 2012-01-19

**Authors:** Jörg Linde, Peter Hortschansky, Eugen Fazius, Axel A Brakhage, Reinhard Guthke, Hubertus Haas

**Affiliations:** 1 Research Group Systems Biology/Bioinformatics, Leibniz Institute for Natural Product Research and Infection Biology- Hans Knöll Institute, Beutenbergstraße 11a, 07745 Jena, Germany; 2 Department of Molecular and Applied Microbiology, Leibniz Institute for Natural Product Research and Infection Biology- Hans Knöll Institute, Beutenbergstraße 11a, 07745 Jena, Germany; 3 Division of Molecular Biology/Biocenter, Medical University Innsbruck, Fritz-Pregl-Str.3, A-6020 Innsbruck, Austria; 4 Friedrich Schiller Univiersity Jena, Jena, Germany

## Abstract

**Background:**

In System Biology, iterations of wet-lab experiments followed by modelling approaches and model-inspired experiments describe a cyclic workflow. This approach is especially useful for the inference of gene regulatory networks based on high-throughput gene expression data. Experiments can verify or falsify the predicted interactions allowing further refinement of the network model. *Aspergillus fumigatus *is a major human fungal pathogen. One important virulence trait is its ability to gain sufficient amounts of iron during infection process. Even though some regulatory interactions are known, we are still far from a complete understanding of the way iron homeostasis is regulated.

**Results:**

In this study, we make use of a reverse engineering strategy to infer a regulatory network controlling iron homeostasis in *A. fumigatus*. The inference approach utilizes the temporal change in expression data after a change from iron depleted to iron replete conditions. The modelling strategy is based on a set of linear differential equations and offers the possibility to integrate known regulatory interactions as prior knowledge. Moreover, it makes use of important selection criteria, such as sparseness and robustness. By compiling a list of known regulatory interactions for iron homeostasis in *A. fumigatus *and softly integrating them during network inference, we are able to predict new interactions between transcription factors and target genes. The proposed activation of the gene expression of *hapX *by the transcriptional regulator SrbA constitutes a so far unknown way of regulating iron homeostasis based on the amount of metabolically available iron. This interaction has been verified by Northern blots in a recent experimental study. In order to improve the reliability of the predicted network, the results of this experimental study have been added to the set of prior knowledge. The final network includes three SrbA target genes. Based on motif searching within the regulatory regions of these genes, we identify potential DNA-binding sites for SrbA. Our wet-lab experiments demonstrate high-affinity binding capacity of SrbA to the promoters of *hapX, hemA *and *srbA*.

**Conclusions:**

This study presents an application of the typical Systems Biology circle and is based on cooperation between wet-lab experimentalists and *in silico *modellers. The results underline that using prior knowledge during network inference helps to predict biologically important interactions. Together with the experimental results, we indicate a novel iron homeostasis regulating system sensing the amount of metabolically available iron and identify the binding site of iron-related SrbA target genes. It will be of high interest to study whether these regulatory interactions are also important for close relatives of *A. fumigatus *and other pathogenic fungi, such as *Candida albicans*.

## Background

A major workflow in Systems Biology is an interlocking circle between experimental and theoretical work [1]. Experimentalists perform high-throughput experiments in order to monitor the response of a biological system to an external stimulus. These data is then used to construct spatio-temporal models from which reasonable hypotheses are generated. These hypotheses are experimentally verified or falsified. Using the results of these experiments, scientists are able to refine the model and thus generate new knowledge [2].

One way of describing biological systems are networks. Networks are graphical representations, where the nodes represent the objects of interest and edges represent relations between these objects [3]. Network models help to explain, understand and describe the functioning of a cell [4]. In many cases we do not know the underlying interaction networks within the system of interest. Network inference aims at the deduction of these networks utilizing high-throughput data and prior knowledge. The inference of gene regulatory networks consists of three parts: the identification of potential regulators, the prediction of target genes, and the inference of the mode of interaction (e.g. activation or repression). A number of approaches are established to perform this task, such as setting up Bayesian Networks [5], information theoretical approaches [6-8], regression based inference [9-11], and differential equation models [12-17]. A number of studies successfully applied these methods for different biological purposes, e.g. modelling of immune diseases [10,13], full genomic models of *Escherichia coli *[8] and *Saccharomyces cerevisiae *[18], and models of pathogenic fungi [16]. It has been shown that the integration of different data sources improves the reverse engineering approach [10,19-21].Since different data sources might be contradictory, it is advantageous to softly integrate them during the modelling procedure. That means, proposed interactions can be scored by the confidence of the prior knowledge source and might be removed if they contradict too much to the measured data. A recent study shows how the Systems Biology circle supports network inference [22]. Due to the large amount of available data and knowledge *E. coli *is best suited as model organism for network inference. However, this task is more difficult for pathogenic fungi by virtue of the small amount of data and small number of known interactions.

*Aspergillus fumigatus *is an airborne saprophytic fungus [23]. Humans constantly inhale numerous conidia of *A. fumigatus*, which are usually eliminated by the immune system. However, in immunocompromised individuals the fungus can cause life-threatening infections [23]. In fact, the number of infections has been dramatically increased due to the growing number of immunocompromised individuals [24-26].

The human host evolved a number of strategies to prevent microbial infection. One important strategy is to keep iron away from the pathogen [27]. Iron is an essential metal required as a cofactor for several proteins, as well as for a number of biochemical processes. However, within the human host, iron is bound to proteins such as haemoglobin, ferritin, transferrin, and lactoferrin. Consequently, there is almost no free iron available [28]. Thus, the acquisition of iron is an important virulence attribute of most pathogens. During co-evolution, *A. fumigatus *has developed a number of efficient iron acquisition pathways: 1) reductive iron uptake, 2) uptake via siderophores, and 3) low-affinity uptake (for a more detailed description see [29]). Since excess of iron is toxic for a cell, iron homeostasis needs to be tightly regulated in *A. fumigatus*. The knowledge about the molecular interactions underlying these regulations is still fragmentary. The transcription factors SreA and HapX have been identified as a counter pair [30-32]. Under iron replete conditions, SreA is activated and represses iron uptake. Under these conditions, SreA also represses *hapX *transcription. Since HapX is a repressor of iron consumption pathways, SreA indirectly activates iron consumption. Moreover, HapX also acts as an activator of iron acquisition. A number of target genes are known for both regulators, however we are still far from a complete understanding of iron homeostasis in *A. fumigatus*.

Recently, we proposed a model predicting regulatory interactions for iron uptake of another fungal pathogen, *Candida albicans*, when the fungus is adhering to and invading into human epithelial cells [17]. The model is based on time series expression data during experimental infection of reconstituted human oral epithelium. The usefulness of these data lies in the fact that it re-samples important parts of a real infection scenario. On the other hand, in the previous modelling approach a number of environmental parameters are not constant during infection, such as pH and nutrient availability. This may have caused side effects and made it difficult to decide whether the proposed interactions are purely based on changes in iron availability or other environmental parameters. Such environmental variations finally hamper experimental verifications of the proposed interactions. The use of *in vitro *time series expression data after a change from iron depleted to iron replete conditions will help to decide which interactions *C. albicans *uses to regulate iron homeostasis. For *A. fumigatus*, such time series expression data is already available and utilized in this study.

In the present work, we propose the first computational model of the regulation of iron homeostasis genes in *A. fumigatus *using high-throughput gene expression time series data after a shift from iron starvation to iron replete conditions [31]. It is based on a set of linear differential equations and utilizes selection criteria such as sparseness and robustness [17,21,33]. Since the soft integration of prior knowledge has been shown to improve the reliability of the predicted networks [10,19-21], our modelling approach softly integrates three kinds of prior knowledge: Northern blot analysis under limited iron [31,32], microarray expression analysis of transcription factor knock-out mutants [31,32], as well as the occurence of transcription factor binding motifs analysis in regulatory regions of genes [31,34-36]. The inferred model predicts new transcription factor to target gene interactions. A recent study utilizes Northern blots and experimentally verifies two of these interactions [37], while another predicted interaction is falsified and one remains unevaluated. Using the results of the recent experiments as additional prior knowledge, we are able to refine our model. The final network model predicts a number of SrbA targets. To study, whether or not the transcriptional regulator directly binds to these target genes, we performed motif searching that lead to the identification of potential SrbA binding sites in the promoters of the predicted target genes. Indeed, wet-lab experiments demonstrate high-affinity binding capacity of SrbA to the promoters of *hapX, hemA *and *srbA*.

## Methods

### Data and imputation

**Figure 1:**
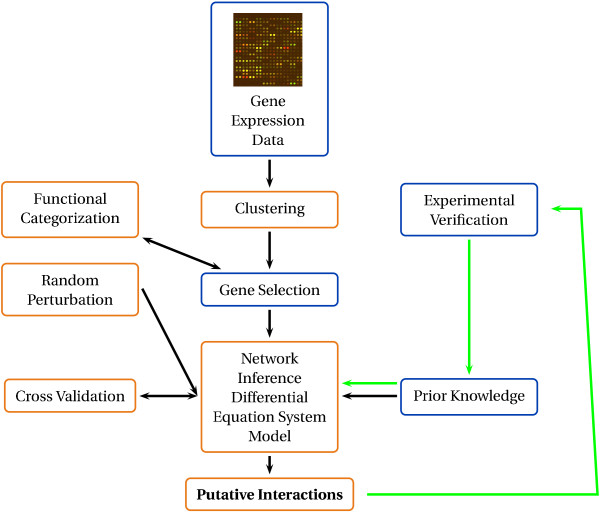
**Overview of applied workflow**. The green lines illustrate how the cyclic workflow of Systems Biology was applied in this study.

Schrettl *et al*. performed full-genomic transcriptional profiling of *A. fumigatus *as response to the change from iron depleted growth to iron replete growth [31]. They monitored gene expression at five timepoints after adding iron to the culture medium: 10 min, 30 min, 60 min, 120 min, 240 min. We used the preprocessed (i.e. normalised and logarithmised) data of Schrettl *et al*. [31]. Figure 1 gives an overview about the applied methods. Since clustering and network inference need complete data, we imputed missing values using the Bayesian Principal Component Analysis (BPCA) imputation from the R-package 'pcaMethods' [38]. This method performed best among a set of different imputation methods (for more information see additional file 1, table S1).

### Clustering

Schrettl *et al*. identified 1147 genes to be differentially expressed within the wild-type strain comparing iron depleted and iron replete conditions [31]. We added *srbA *to this set (see candidate genes for regulatory network model) and collected (imputed) expression values of these genes. We applied fuzzy c-means clustering [39] to this expression matrix. The optimal number of clusters was estimated as previously described [13,17]. In short, 42 cluster validity indices (Dunn's index and the Davis-Bouldin index with 18 generalizations each as well as the silhouette width and five other indices as described in [13]) capturing different aspects of a clustering structure were used to assess the partitions based on 2 up to 20 clusters. The number of clusters that was ranked best by the most validity indices was chosen.

### Overrepresented gene ontology terms

In order to identify key biological processes/functions most significantly enriched with genes within each of the clusters, we performed functional categorization and identified significantly overrepresented categories using the tool FungiFun [40]. We applied both Funcat [41] (all four hierarchical levels) and Gene Ontology [42] (Biological Process and Molecular Function) categorization.

### Network prediction

Network inference was performed similarly as previously described [17] applying the Net *Gene*rator tool [33]. This tool is available upon request. In short, the network inferences approach has the following features:

1. It is based on a set of linear differential equations and models the temporal change of the expression intensity *x*_*i*_(*t*) of gene *i *(*i *= 1..*n*) at time *t *as the weighted sum of the expression intensities of all other genes and an external stimulus *u*(*t*) at time *t *(see equation 1). The external stimulus *u*(*t*) is modelled as a stepwise constant function representing the change from iron depletion to iron repletion.

(1) $${ẋ}_{i}\left(t\right)=\overset{n}{\underset{j=1}{\sum }}w_{i,j}x_{j}\left(t\right)+b_{i}u\left(t\right)$$

2. Based on the given time series data, the tool calculates the gene regulatory matrix *W *and the perturbation vector *B*. The parameter *w*_*i,j *_(component of *W*) represents an influence of gene *j *on the expression of gene *i*, while the parameter *b*_*i *_(component of *B*) represents the impact of the external stimulus given by the function *u*(*t*). Non-zero parameters define the edges of the regulatory network. A positive parameter *w*_*i,j *_denotes an activation and a negative parameter denotes a repression of gene *i *by gene *j.*

3. The approach follows the selection criterion of sparseness. Using a heuristic search strategy it tries to minimise the number of non-zero parameters(interactions) which are necessary to fit to the measured data points.

4. The approach follows the selection criterion of robustness, i.e. technical noise in measured mRNA concentrations caused by the microarray technology does not alter inferred regulatory interactions. This is achieved by iterating the network inference procedure 1000 times using randomly perturbed input time series data (Gaussian noise with mean 0 and standard deviation 0.05 added to the measured and pre-processed data) [13,16]. Only edges which are confirmed by more than 50% of the iterations are considered to be robust.

5. The inference approach uses prior knowledge (i.e. putative regulatory interactions based on additional data to time series expression data). Based on the confidence of the prior knowledge source, it is possible to score each proposed interaction. Since different data sources might be contradictory, it is advantageous to softly integrate them during the modelling procedure. If a proposed interaction contradicts the measured data too much it might be removed. If necessary, the tool adds new interactions not covered by the prior knowledge in order to fit to the measured data.

6. Interactions included in the regulatory model might mainly be based on their occurrence in the set of prior knowledge, rather than on the expression data. Thus, we tested whether or not the predicted interactions are robust against changes in the set of prior knowledge by iterating the modelling approach 1000 times while randomly skipping 10% of all interactions in the set of prior knowledge in each run. Again, only edges which are confirmed by more than 50% of the iterations are considered to be robust.

Three different sources are used to compile prior knowledge for the prediction of gene regulatory networks:

**Source 1**: Evidence of transcription factor - target gene interactions based on single experiments (e.g. Northern blots). Confidence score = 0.5

**Source 2**: Gene expression studies under limited iron conditions and expression analysis of transcriptional regulator knock-out mutants. Confidence score = 0.25

**Source 3**: Occurrence of the respective transcription factor binding motif in the upstream intergenic regions of iron homeostasis genes. Confidence score = 0.125.

The score is additive, i.e., if an interaction is predicted by several sources the used score equals the sum over all confidence scores for the respective sources.

### SrbA binding site

Three DNA sequences with high binding-affinity to the transcriptional regulator Sre1 were identified in *Schizosaccharomyces pombe *[43]. Sre1 and SrbA show high sequence similarity. Furthermore, the SrbA protein contains a basic helix-loop-helix/leucine zipper (bHLHZ) domain. This domain has been shown to specifically bind DNA in *S. pombe *[43]. The three high-affinity Sre1 binding-sites are characterised by a conserved ATC at the 5' end and a conserved AT at the 3' end, while the remaining parts are highly variable (5'-ATCNNNNNAT-3'). For the human ortholog of Sre1 and SrbA, the adenosin and thymidin enable the contact with the protein [44]. To predict a SrbA binding site in *A. fumigatus*, we first downloaded intergenic regions of genes predicted to be SrbA targets by our network model. Next, these intergenic regions were scanned for the occurrence of the three high-affinity binding sites of Sre1 allowing maximal two mismatches [45]. Finally, we only considered those sites which contain the conserved 5' and 3' AT.

To determine whether *A. fumigatus *SrbA recognizes the identified putative binding sites, the bHLHZ domain of SrbA (amino acids 161-267,"SrbA161-267" ) was produced in *E. coli *and purified. The protein domain was analysed by real-time *in vitro *surface plasmon resonance (SPR) binding assays. Immobilized DNA duplexes (see additional file 2 for experimental details ) were used to test whether or not the protein domain can bind to the predicted DNA sites.

## Results

### Clustering and overrepresented GO categories

The BPCA (Baysian Principal Component Analysis) method gave best results for imputation (see additional file 1, table S1) and was thus chosen to impute missing values into the original gene expression data set.

**Figure 2:**
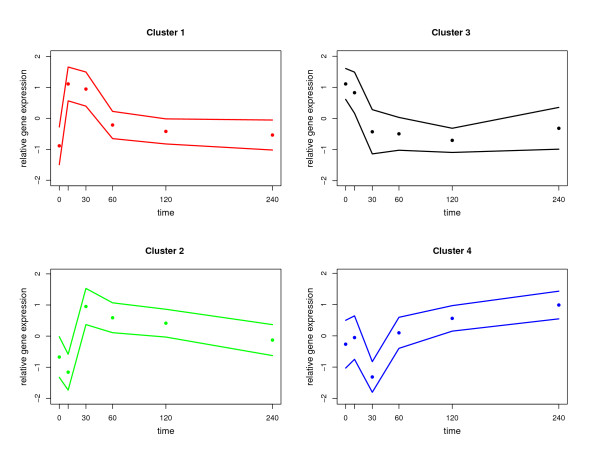
**Cluster analysis results**. The best partition consists of four clusters. Points: mean (logarithmised, scaled and centred) expression values of all genes in the cluster, lines: standard deviation.

The optimal number of clusters for partitioning the expression data was found to be four (see additional file 3, figure S3). Scaled time series profiles are visualised in figure 2. Additional file 4 shows to which cluster each gene belongs to, while additional file 5 lists significantly overrepresented categories for the respective clusters.

Cluster 1 consists of genes which show a quick up-regulation after adding iron to the culture medium. This cluster is significantly enriched with genes involved in iron-dependent processes including iron-sulfur cluster biosynthesis, heme biosynthesis, respiration, TCA cycle assembly as those categories are overrepresented according to Funcat (level 2-4) and to GO-Biological Process (GOBP). Furthermore, cluster 1 is significantly enriched with genes involved in transcriptional regulation. In fact, 17 genes belong to the GO category "transcription" (p = 0.0028). Among them are important regulators of iron homeostasis, namely the transcription factors SreA and PacC. Since the averaged expression profile in this cluster shows a quick up-regulation, further so far unknown regulators might be involved in regulating genes involved in iron homeostasis.

Cluster 2 also shows a quick, but delayed, up-regulation. This cluster mainly consists of genes involved in RNA processing.

Cluster 3 consists of genes which are highly expressed under iron starvation conditions and are therefore down-regulated after adding iron. Interestingly, all 49 genes belonging to the SreA regulon [31] are members of cluster 3, showing that the clustering partitioned the genes into biologically relevant groups.

The majority of the characterized SreA target genes are involved in iron uptake, including reductive iron assimilation and siderophore mediated iron acquisition (Funcat level four). Another SreA target is the HapX-encoding gene. Taken together, these data demonstrate that Cluster 3 encodes the major genes required for adaptation to iron starvation.

Cluster 4 mainly consists of genes involved in oxidation-reduction processes (GOBP). Its mean expression profile is characterized by a local minimum after 30 minutes followed by an up-regulation.

Taken together, the best characterized gene sets are found in Cluster 1 with iron-dependent pathways and Cluster 3 with pathways that are important for adaptation to iron starvation. The co-clustering of many genes with yet unknown functions indicates similar features.

### Candidate genes for regulatory network model

In order to model a network, it is necessary to select a set of relevant genes, which will be represented by nodes in the network model. Table 1 summarises information about genes which are included in the network model. From each cluster, we chose a number of genes involved in important parts of iron homeostasis system, or genes coding for regulators of iron homeostasis.

**Table 1:** Model genes

ID	Name	Cluster	Function
AFUA 5G11260	SreA	1	siderophore transcription factor SreA
AFUA 5G03920	HapX	3	bZIP transcription factor (HapX), putative
AFUA 3G11970	PacC	3	C2H2 transcription factor PacC, putative
AFUA 2G01260	SrbA	1	HLH transcription factor, putative
AFUA 5G06270	HemA	1	5-aminolevulinic acid synthase
AFUA 5G10370	Sdh2	1	succinate dehydrogenase iron-sulphur protein
AFUA 4G14640	Fet4	2	low-affinity iron transporter, putative
AFUA 5G10610	AFUA 5G10610	2	ubiquinol-cytochrome c reductase iron-sulfur subunit precursor
AFUA 3G03640	MirB	3	siderochrome-iron transporter (MirB), putative
AFUA 5G03800	FtrA	3	high-affinity iron permease CaFTR2
AFUA 1G04450	SidL	4	siderophore biosynthesis protein, putative
AFUA 1G07480	Hem13	4	coproporphyrinogen III oxidase, putative

Genes used as candidates for the regulatory network model.

As they are important regulators, SreA and HapX are parts of the model. Another regulator is PacC, which has been shown to be involved in regulating siderophore biosynthesis genes in the close relative *Aspergillus nidulans *[46]. Moreover, its *C. albicans *orthologue Rim101 might be involved in regulating iron acquisition genes during (experimental) oral infection [17]. Finally, we included the regulator SrbA because recent data suggest that it might be involved in regulating the metabolically available iron level. The *C. neoformans *orthologue is essential for growth under iron starvation and regulates reductive iron uptake [47]. In *A. fumigatus*, the protein is activated under ergosterol-limited conditions and might be involved in activating iron uptake [48]. Finally, expression of *srbA *is downregulated under iron replete conditions [31]. However, the fold-change of *srbA *is slightly smaller than the cut-off applied by Schrettl *et al*. [31] for the identification of differentially expressed genes.

In order to have genes coding for proteins involved in different parts of iron homeostasis, we included a low-affinitiy iron transporter (Fet4, cluster 3), one high-affinity iron transporter (FtrA, cluster 3), one protein involved in siderochrome-iron transport (MirB, cluster 3) and one protein involved in haem uptake (HemA, cluster 1). Furthermore, we included genes coding for proteins involved in siderophore and haem biosynthesis (SidL, Hem13, cluster 4), as well as genes coding for iron-sulfur cluster proteins (Sdh2 cluster 1 and AFUA 5G10610 cluster 2). Note, that we only used one imputed value for network inference, namely the expression value of *sreA *at timepoint zero.

### Prior knowledge

Table 2 lists the prior knowledge used in this study.

**Table 2:** Prior knowledge

Regulator	Target	Interaction	Source	Score	Reference
HapX	*sreA*	repression	1	0.5	[29]
HapX	AFUA_5G10610	interaction	2	0.25	[32]
HapX	*mirB*	activation	1,3	0.625	[32,51]
HapX	*hemA*	repression	2	0.25	[32]
HapX	*sdh2*	repression	2	0.25	[32]
input	*sreA*	activation	2	0.25	[31]
input	*hapX*	repression	2	0.25	[32]
PacC	*mirB*	interaction	3	0.125	[46]
PacC	*ftrA*	interaction	3	0.125	[46]
PacC	*hem13*	interaction	3	0.125	[46]
SreA	*sdh2*	repression	2	0.25	[31]
SreA	*hemA*	repression	2	0.25	[31]
SreA	AFUA_5G10610	repression	2	0.25	[31]
SreA	*hapX*	repression	1,3	0.625	[31]
SreA	*mirB*	repression	1	0.5	[31]
SreA	*ftrA*	repression	1,3	0.625	[31]
SreA	*sidL*	repression	3	0.125	[31]

SrbA	*hapX*	activation	1	0.5	[37]
SrbA	*ftrA*	activation	1	0.5	[37]
SrbA	*mirB*	activation	1	0.5	[37]
SrbA	*hem13*	activation	1	0.5	[37]
SreA	*hem13*	no interaction	1	0.5	[37]

The table lists regulator-target gene interactions, the type of interaction, the source of prior knowledge, the score used and references. The second part of the table lists additional prior knowledge we exploited in order to refine the model within the second round of modelling. "Input" denotes the fact that a target gene is regulated by the external perturbation (shift from iron replete to iron depleted conditions).

As prior knowledge source 1, we used Northern blot results of transcription factor knock-out mutants comparing iron replete conditions with iron depletion. In that way, we identified two potential target genes of HapX [29,32] and three for SreA [31]. As prior knowledge source 2, we made use of the full genomic gene expression profiles of transcription factor knock-out mutants, i.e. we added a potential interaction if a gene is differentially expressed in the knock-out mutant compared to the wild type. Altogether, we identified eight potential interactions this way [31,32].

For prior knowledge source 3, we added a potential interaction if the respective binding motif of a transcription factor occurred in the regulatory region of genes included in the model. For SreA we used the consensus binding motif 5'-ATCWGATAA-3' [31] which we found in three potential target genes. The PacC consensus binding motif 5'-GCCARG-3' of the *A. nidulans *[46] was also found in three potential target genes. HapX interacts with the 5'-CCAAT-3' binding box in *A. nidulans *[30]. In order to test whether or not this interaction also occurs in *A.fumigatus*, we proposed one gene with the CCAAT-box in their promoters as HapX targets.

### Regulatory network of iron homeostasis genes

**Figure 3:**
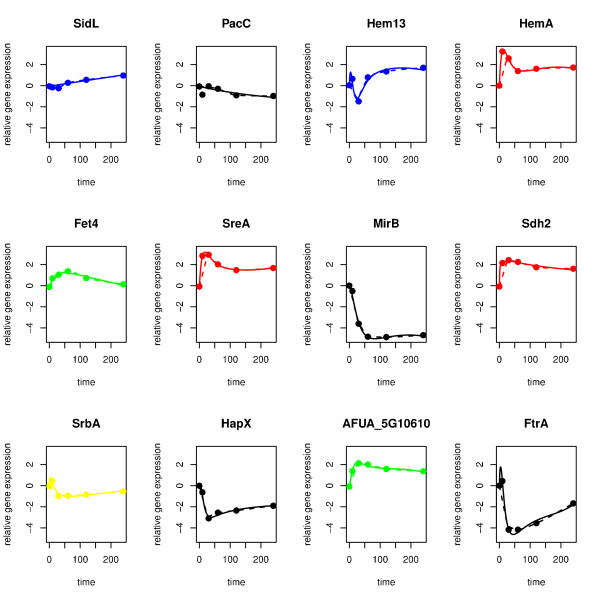
**Data fit for the initial model**. Data fit of the initial model (i.e. before refining the prior knowledge and before testing for robustness). Dots: measured data points, dashed lines: interpolated data points, solid lines: model-simulated data points.

**Figure 4:**
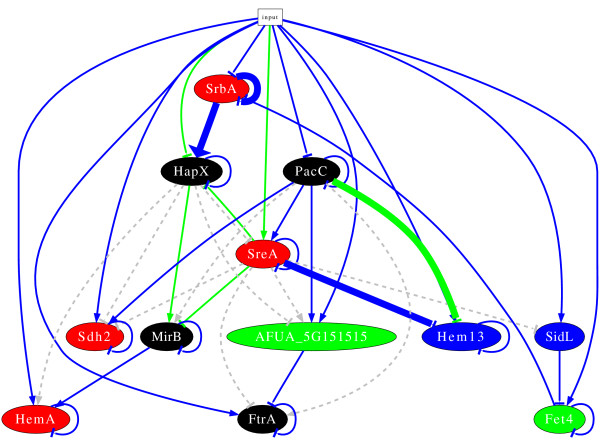
**Inferred regulatory network before refinement of the prior knowledge**. The node colour denotes the cluster the gene belongs to. An edge between two nodes represents an interaction; arrows are activations while bars are repressions. Green edge: based on measured kinetics, consistent with prior knowledge, and robust(found more than 50% in models based on randomized input time series data and cross-validation of prior knowledge); blue edge: not in prior knowledge, based on measured kinetics, and robust; Grey edge: predicted by the prior knowledge but contradicting time series data, not in the model.

Following the modelling approach described in the methods chapter, we inferred two regulatory network models. The first model is based on the prior knowledge being available previous to the study of Blatzer *et al*. [37]. Figure 3 shows that the initial model (i.e. before tested for robustness) fits well to the measured kinetics. Figure 4 presents the network after removing non-robust edges. The model explains how SreA and HapX interact to regulate iron homeostasis genes and elucidates the role of further regulators. The model predicts the repression of *hapX *by SreA, which is supported by the SreA binding site in the upstream region. As expected, HapX itself does not repress *sreA*, even though this interaction is supported by source 3. This interaction only takes place under iron deplete conditions [29]. The known repression of *mirB *by SreA [32] was confirmed by our model. This shows that our modelling approach is capable of finding de-novo biologically relevant interactions. Table 3 summarizes target genes of transcriptional regulators newly predicted by the model. Of high interest is the activation of *hapX *by SrbA. If sufficient amount of metabolic iron is available, SrbA might down-regulate iron consumption by activating *hapX*.

**Table 3:** Transcriptional regulators and predicted target genes

Regulator	newly predicted target genest	
	Before refinement	After refinement

SreA	sreA, hem13*	*sreA*

HapX	*hapX*	*hapX*

PacC	*pacC, hem13*	

SrbA	*srbA,****hapX***	***srbA,hemA***

For each regulator, newly predicted target genes are shown. A target gene is counted as "newly predicted" if it was predicted by the time series data without prior knowledge, or if it was predicted by prior knowledge based on the occurrence of transcription factor binding motif (source 3) and was found to be consistent with the gene expression data. The second column of the table lists the results after refining the model. Bold genes have been validated by Northern blots [37] (before refinement) or binding to SrbA (after refinement). The interaction marked with asterix was falsified.

Similar to *A. fumigatus*, the *C. neoformans *SrbA ortholog activates genes involved in high-affinity iron uptake of iron, including genes in both siderophore-mediated and reductive iron transport, as well as heme biosynthesis [47]. Moreover, heme biosynthesis, including Hem13, is activated by the SrbA orthologs in *S. pombe *and *C. neoformans *[43,47]. Since most oxygen-dependent enzymes are also iron/heme-containing, the iron starvation response is often coordinately regulated with the response to hypoxia [49]. Therefore, the cellular needs for oxygen and iron are tightly linked, which most likely provides the rational for coregulation of iron, heme and oxygen metabolism by SrbA in *A. fumigatus*.

Furthermore, the model newly predicts two regulators of *hem13*. PacC and SreA are predicted to down-regulate this gene under iron deplete conditions. Finally, the model predicts a number of self-repressing interactions. These self-repressing interactions can be interpreted as degradation of the mRNA.

### Experimental verification

In a recent study, Blatzer *et al*. [37] performed gene expression analysis under iron replete and iron depleted conditions of a number of genes involved in iron homeostasis using Northern blots. The special focus of this study is the regulatory role of SrbA. Results of the study verify the activation of *hapX *by SrbA which was predicted by our first model (see figure 4). This shows that our modelling approach is not only able to include current knowledge but also correctly predicts interactions. Note, that no prior knowledge was used to predict this interaction. Another predicted interaction was falsified by the experimental results [37]. It turns out from the Northern blot analysis that there is no evidence that SreA represses *hem13*. Instead, the experimental study indicates an activation of *hem13 *by SrbA. The interaction between PacC and *hem13 *was not experimentally tested.

### Refining the network model

Having the experimental results of the recent study at hand [37], we were able to complete the prior knowledge in order to refine the network model. Table 2 (second part) lists the interactions additionally included as prior knowledge in the next modelling round. From a methodological point of view, this modelling round followed exactly the same strategy as the first one, i.e. we identified interactions which are robust against technichal noise in the mRNA concentration and do not change when we randomly skipping parts of the prior knowledge (see methods).

**Figure 5:**
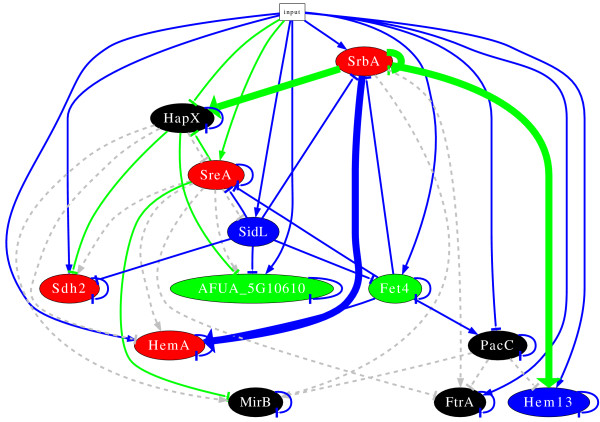
**Inferred regulatory network after refinement of prior knowledge**. For description see figure 4.

Figure 5 shows a visualization of the model. Additional file 6, table S6 displays results of the resampling by perturbation of input data and cross-validation. Alternative configurations of the resampling were studied showing similar results as presented (data not shown). Due to the lack of a "gold standard" for the "true" network we are unable to decide about the best configuration of resampling.

The questionable interactions predicted by the first model do not occur in this model any more. In the new model, *hem13 *and *hapX *are activated by SrbA, which is in agreement with the new prior knowledge [37]. By contrast, the activation of *ftrA *and *mirB *by SrbA was not found. Instead, the refined model predicts a direct activation of *hemA *by SrbA. This activation was already predicted before refinement of the model, but the activation was indirectly indicated in figure 4.

### SrbA binding site and regulon

The refined network model consists of four target genes for SrbA. Two of them, *hapX *and *hem13*, have been validated by Northern blots [37], while the self-repression of *srbA *and the activation of *hemA *remain untested. In order to test these two interactions and to see if the Northern blots findings are a result of a physical interaction, we predicted SrbA binding sites using motif searching (see methods). For experimental verification, we used real-time *in vitro *surface plasmon resonance (SPR) binding assays of the predicted binding sites and the purified SrbA-DNA-binding-domain (see methods and additional file 2 for details and figures). Table 4 summarises the results. The predicted binding site within the *srbA *promoter (-735 to -726) has no mismatch when aligned to the consensus sequence of high-affinity sites in *S. pombe*. *A. fumigatus *SrbA161-267 shows high-affinity DNA-binding responses that fit to a *K*_*D *_value of 0.63*nM *for this binding site. By contrast, only low-affinity binding (*K*_*D *_> 300*nM*) was observed for a DNA duplex mutant that served as negative control and altered the *srbA *binding motif from ATCATACGAT to ATATAACATA. Furthermore, high affinity SrbA161-267 binding was observed as well with putative binding sites that had only one mismatch compared to known binging sites in *S. pombe*. Kinetic binding responses on duplexes encoding *hapX *(-1340 to -1331) and *hemA *(-527 to -518) promoter regions fit with *K*_*D *_values of 4.6*nM *and 4.2*nM*, respectively. Additionally, we identified two sites with weak binding in *hapX *and *hemA*, respectively. Taken together, SrbA has high-affinity binding capacity to binding sites in *hapX, hemA *and *srbA*. Together with our predicted network and the Northern blot analyses [37], these results suggest a direct physical interaction of SrbA with these target genes. The consensus of the high-affinity sites is 5'-ATC[G--A][T--G][A--G][C--T][G--C]AT-3'. We used the experimentally validated sites to scan for further putative SrbA target genes within the *A. fumigatus *genome, allowing up to 2 mismatches in the variable region (see additional file 7, table S7). About 13% of the *A. fumigatus *genes contain one of these sites with maximal one mismatch in their regulatory region. A functional categorisation of this gene list resulted in overrepresented functional categories such as "Heavy metal binding (Cu, Fe, Zn)" and "Lipid, fatty acid and isoprenoid metabolism". This adds evidence to the hypothesis that SrbA links ion concentration and fatty acid metabolism.

**Table 4:** SrbA binding sites

Gene	Position	Strand	Sequence	*k*_*a*_(*M*^-1^*s*^-1^)	*k*_*d*_(*s*^-1^)	*K*_*D*_(*nM*)
*srbA*	-735 to -726	Sense	ATCATACGAT	1.48 ± 0.05 * 10^7^	9.27 ± 0.29 * 10^-3^	0.63 ± 0.04
srbA*	-735 to -726	Sense	ATATAACATA	5.92 ± 0.08 * 10^5^	2.29 ± 0.01* 10-1	386 ± 7.3
*hapX*	-774 to -783	Antisense	ATCCTCCCAT	5.68 ± 0.07 * 10^5^	1.61 ± 0.01* 10-1	282 ± 5.1
*hapX*	-1340 to -1331	Sense	ATCAGATGAT	3.10 ± 0.01 * 10^7^	1.44 ± 0.01 * 10^-1^	4.64 ± 0.03
*hemA*	-527 to -518	Sense	ATCGGATCAT	1.41 ± 0.07 * 10^7^	5.88 ± 0.28 * 10^-2^	4.18 ± 0.41
*hemA*	-338 to -347	Antisense	ATCGCCTCAT	1.03 ± 0.01 * 10^6^	3.66 ± 0.02 * 10^-2^	35.7 ± 0.52

The binding of SrbA to five predicted binding sites was analysed. Results indicate three high-affinity binding sites in promoter regions of *hapX, hemA *and *srbA*. The asterix denotes a mutated binding site. Mutated nucleotides are underlined. *k*_*a*_= association rate constant, *k*_*d *_= dissociation rate constant, *K*_*D *_= *k*_*d*_*/k*_*a *_equilibrium dissociation constant.

## Discussion

In this study, we propose a modelling approach based on a set of ordinary differential equations. Even though this approach fits well to the measured time series data, it has the drawback that it is inappropriate for large-scale modelling. In general, a large number of genes being part of a model leads to a large number of parameters to be identified, which may result in over-fitting of the data. Our modelling approach aims at inferring a sparse network (i.e. many parameters are zero) and makes use of resampling techniques where the data are perturbed in a random manner (see chapter "Network prediction" point 4 and 6). Both attempts help to prevent over-fitting. Furthermore, we restrict the number of genes, thereby leading to a smaller number of parameters to be identified. The selection of those genes that are included in the model are directed from experimental findings. However, some additional genes which might be involved in iron homeostasis are not included in the model. Furthermore, there is a number of so far uncharacterised genes which might play a role. The clustering of the expression data helps to get an idea about those genes. One gene of each cluster in the proposed regulatory model could be thought of a cluster representative. In this way, regulatory interactions inferred by our model might be transferred to other pairs of genes belonging to the respective clusters. With the knowledge of co-expression patterns and the regulatory influences proposed by our model, it might be possible to obtain an idea about the function of so far uncharacterised genes.

The modelling strategy makes use of prior knowledge. The cross-validation procedure helps to prevent the model from adapting too much to the given knowledge. However, the prior knowledge incooperated into the model could be changed. In general, we could add more interactions when making use of knowledge based on other organisms. It remains to find out what organisms could be used for this task, i.e. what the maximal evolutionary distance of an organisms that could be used as prior knowledge source is. This also relates to the question of how we chose the local scores for each interaction. The scoring sheme used in this study was already successfully applied [17]. However, when including information from an evolutionary distant organism this scoring scheme needs to be expanded. For example, Northern blot analysis revealed that *mirB *is activated by PacC under alkaline conditions in *A. nidulans *[46]. This would add another category of prior knowledge ("Northern blots of close relatives"). Our models do not predict this interaction, even though we use prior knowledge based on the occurrence of the PacC binding site.

A recent study shows that sidL is expressed independently of SreA [50]. Our models predict no interaction between these genes, even though it was proposed by the prior knowledge source 3. This shows that our modelling strategy is not blindly adapting to the given set of prior knowledge. If an interaction proposed by the prior knowledge contradicts the measured expression data too much, our modelling approach removes it from the predicted network.

The applied scoring scheme assigns the highest score for prior knowledge based on Northern blots. The rationale behind this is that Northern blots are no high-throughput experiments and thus we believe these experiments give strong evidence that the respective regulatory interactions exist. However, the Northern blots were performed at steady state (24 hours after adding iron). On the other hand, the used expression data focused on early effects after the change to iron replete conditions (10 to 240 minutes). This might explain some discrepancies between the Northern blot data and the proposed models.

Here, we report the first SrbA binding sites in *A. fumigatus*. The data revealed a remarkable sequence similarity between *S. pombe *and *A. fumigatus*. An interesting future task will be to identify further SrbA target genes and to analyse whether the defined binding sites are conserved throughout other fungal species. All high-affinity binding sites of *S. pombe *and *A. fumigatus *show a conserved C at the third position. While the existence of the conserved AT can be explained by the fact that the human SrbA ortholog physically interacts with these nucleotides, the reason for the conservation of the C remains to be elucidated.

## Conclusions

This study demonstrates how the Systems Biology circle is carried out, i.e. how experimental work and modelling iteratively interact, in order to gain understanding of a biological system. Analysing gene expression time series data and using a modelling approach based on a set of differential equations, we were able to predict new regulatory interactions controlling iron homeostasis in *A. fumigatus*. Wet lab experiments proved that the proposed modelling approach allows to predict novel biologically relevant interactions. Results of the latest experiments were used to refine the predicted model. Taken together, this underlines that using prior knowledge during network inference improves the prediction quality of the reverse engineering. Together with the experimental results, we identified a new iron homeostasis regulatory network based on the amount of metabolically available iron. Furthermore, we found that SrbA physically interacts with its predicted target genes via specific DNA-binding and identified the SrbA binding site in *A. fumigatus*.

In a previous study, we predicted a regulatory network concerning iron acquisition by the fungal pathogen *C. albicans *during an experimental infection. This model was based on a similar modelling strategy, i.e. it also exploits gene expression data and uses a set of prior knowledge. In the case of *C. albicans *it remains unclear which of the predicted regulatory interactions is exclusively based on limited iron. In contrast, for *A. fumigatus *we do not know which of the (predicted) interactions play a role in an *in vivo *infection process. Further experiments will focus on time series expression of *A. fumigatus *in an (experimental) infection and on expression data of *C. albicans *under *in vitro *iron limitation. It will be interesting to figure out whether *C. albicans *also regulates iron homeostasis based on the amount of metabolically available iron. This will give us the opportunity to compare regulations of iron homeostasis for both important fungal pathogens. Together with the growing amount of available expression data for both fungi we will be able to expand our models to other important processes, thus making *A. fumigatus *and *C. albicans *model organisms for fungal infections.

## Authors' contributions

HH and RG directed the study. JL carried out the analyses of the data, performed the modelling, and wrote the manuscript supported by the coauthors. HH assisted in choosing candidate genes for the model. EF and JL scanned for the SrbA binding sites. PH designed and performed the experiments for the binding site. HH, PH and AB assisted in the interpretation of the results. All authors read and approved the final manuscript.

## Supplementary Material

Additional file 1 **Table S1 - Imputation**. The whole genome expression data (wild-type and mutant) includes 20.4% missing values. Since clustering and network inference need complete observations, we imputed those missing values following a similar approach applied by Albrecht *et al*. [52]. First, we removed 1253 genes (rows), which had 100% missing values (genes not spotted on the chip). Then, we tested the following imputation methods. From the R package 'impute' [53] K-nearest-neighbour; from the R package 'pcaMethods' [38]: probabilistic Principal Component Analysis (PCA), Bayesian PCA (BPCA), Single-Value-Decomposition impute (SVD impute), PCA by non-linear iterative partial least squares (NIPALS), Neural network based non-linear PCA (NLPCA), and Local Least Squares (LLS) imputation. The concatenation of the wild-type data and the mutant data was used together, since more data improves imputation. For test purpose we found the largest sub-matrix, which consists of full observations (5566 genes with no missing data) and constructed a test-data matrix by randomly introducing artificial missing values in this sub-matrix, keeping the distribution of missing values within the columns the same like in the original matrix. We used the different imputing methods on the test-data and compared the results to the original data in terms of the root mean square error (RMSE). In the first step, we ran each method separately on a range of parameter settings to identify optimal local parameter values. In the second step, we applied the methods using the respective optimal parameter settings on 500 random test matrices. Finally, we compared the methods to each other using the mean RMSE values. The table summarizes results of both steps. Click here for file

Additional file 2 **SrbA binding site**. Experimental details and results of real-time *in vitro *binding analysis of SrbA. Click here for file

Additional file 3 **Figure S3 - Cluster validity index**. Validity indeces for partitions based on two up to twenty clusters are shown. The maximum denotes the best partition. Click here for file

Additional file 4 **Table S4 - Cluster annotation**. This table shows to which cluster each gene belongs to. Functional annotations and GO annotations are given. Click here for file

Additional file 5 **Table S5 - Overrepresented functional categories for each cluster**. Overrepresented categories for each cluster. The file consists of 20 sheets. For each cluster, significantly (*p *< 0.01) overrepresented functional categories for Funcat level 1-4 and Gene Ontology are presented. Click here for file

Additional file 6 **Table S6 - Inferred interaction network based on final prior knowledge**. The table summarise results of the inferred network based on the final prior knowledge list. It gives the number of resampling during random perturbation of time series data and during the cross-validation of prior knowledge. Click here for file

Additional file 7 **Table S7 - SrbA regulon**. The table lists *A. fumigatus *genes having the experimentally validated SrbA binding sites in their regulatory region. Click here for file
