# Supplementary material for: Regulatory interactions for iron homeostasis in Aspergillus fumigatus inferred by a Systems Biology approach
Source: BMC Syst Biol. 2012 Jan 19;6:6. doi: 10.1186/1752-0509-6-6 (PMC3305660; doi:10.1186/1752-0509-6-6)
Supplement: Additional file 2 — SrbA binding site. Experimental details and results of real-time in vitro binding analysis of SrbA. [file 1752-0509-6-6-S2.DOC]

# Regulatory interactions for iron homoeostasis in *Aspergillus*

# *fumigatus* inferred by a Systems Biology approach

# Experimental details and results of real-time *in vitro* binding analysis of SrbA.

# Materials and Methods

# *E. coli* expression and purification of recombinant SrbA protein

The sequence coding for SrbA residues 161-267 was amplified by PCR as *Bam*HI-*Hin*dIII fragment using *A. fumigatus* cDNA as template and inserted into a modified *Bam*HI-*Hin*dIII-digested pET-42b plasmid (Novagen), having a N-terminal GST-tag and a cleavage site for tobacco etch virus (TEV) protease.

GST-SrbA161-267 fusion protein was produced by autoinduction in *E. coli* Rosetta 2 (DE3) cells grown at 26°C in 1 l Overnight Express Instant TB Medium (Novagen). 17 g wet cells were collected by centrifugation, resuspended in 200 ml lysis buffer (20 mM NaH_2_PO_4_, 150 mM NaCl, 1 mM AEBSF, pH 7.5) and disrupted using an Emulsiflex C5 high pressure homogenizer (Avestin). Cleared cellular extracts were applied to a GSH Sepharose 4FF (GE Heathcare) column and GST-SrbA161-267 was eluted with 50 mM Tris/HCl, 10 mM glutathione, pH 8.0. After digesting GST-SrbA161-267 by adding 4 μg TEV protease per mg fusion protein at room temperature for 2 hours and a subsequent buffer exchange step, GST was captured by reloading the GSH Sepharose 4FF column. Unbound SrbA161-267 was purified to homogeneity on a HiPrep Heparin FF (GE Heathcare) column that was equilibrated with 50 mM Tris/HCl, 150 mM NaCl, 10% (v/v) glycerol, pH 8.0, followed by elution with a gradient up to 2 M NaCl.

# Surface plasmon resonance binding assays

Real-time analysis were performed on a Biacore 2000 system at 25 °C and data were processed with the evaluation software version 4.1 (GE Healthcare). DNA duplexes encoding putative SrbA binding sites were produced by annealing complementary oligonucleotides using a 5-fold molar excess of the non-biotinylated oligonucleotide (Table 1). The dsDNA was injected on flow cells of a streptavidin (Sigma)-coated CM3 sensor chip at a flow rate of 10 µl/min until 65 to 70 RU had been bound. SrbA161-267 was injected in running buffer (10 mM HEPES pH 7.4, 150 mM NaCl, 3 mM EDTA and 0.005% (v/v) surfactant P20) at concentrations from 3.13 to 100 nM (calculated for the homodimer). Sample injection and dissociation times were set to 200 and 400 seconds at a flow rate of 30 µl/min. Each injection was performed 3 times. Regeneration was achieved with 10 mM Tris/HCl pH 7.5, containing 0.5 M NaCl, 1 mM EDTA and 0.005% (w/v) SDS for 1 minute. Refractive index errors due to bulk solvent effects were corrected with responses from non-coated flow cell 1 as well as subtracting blank injections. Dissociation constants were calculated from the kinetic rate constants for SrbA-DNA complex formation and dissociation derived from a 1:1 interaction model including a mass transport term.

# Results and Discussion

# SrbA binds to target gene promoters

Transcription of cholesterol and fatty acid biosynthesis genes is controlled by Sterol regulatory element binding proteins (SREBPs). These hypoxic transcription factors are required for adaptation to a low-oxygen environment and belong to the class of basic region/helix-loop-helix/leucine zipper (bHLHZ) transcription factors. An unique feature of SREBPs is the presence of an atypical tyrosine residue in their basic regions that allows for specific binding to sterol regulatory elements (SREs). Human SRBP-1a targets a non-palindromic SRE-1, 5´-ATCACCCCAC-3´ (Parraga et.al., 1998) and *S. pombe* Sre1p showed high affinity binding to SREs from the promoter regions of *sre1*+, *hem13*+ and *erg3*+ (Todd et.al., 2006). The consensus sequence from the latter 3 *S. pombe* SREs (5´-ATCRBHCSAT-3´) was used for an *in silico* identification of putative SREs in the promoter regions of putative SrbA target genes in *A. fumigatus*.

To determine whether *A. fumigatus* SrbA could recognize the identified putative SREs, we expressed and purified the bHLHZ domain of SrbA (amino acids 161-267) from *E. coli* (Fig. 1) and performed real-time *in vitro* surface plasmon resonance (SPR) binding assays of this recombinant protein to immobilized DNA duplexes.


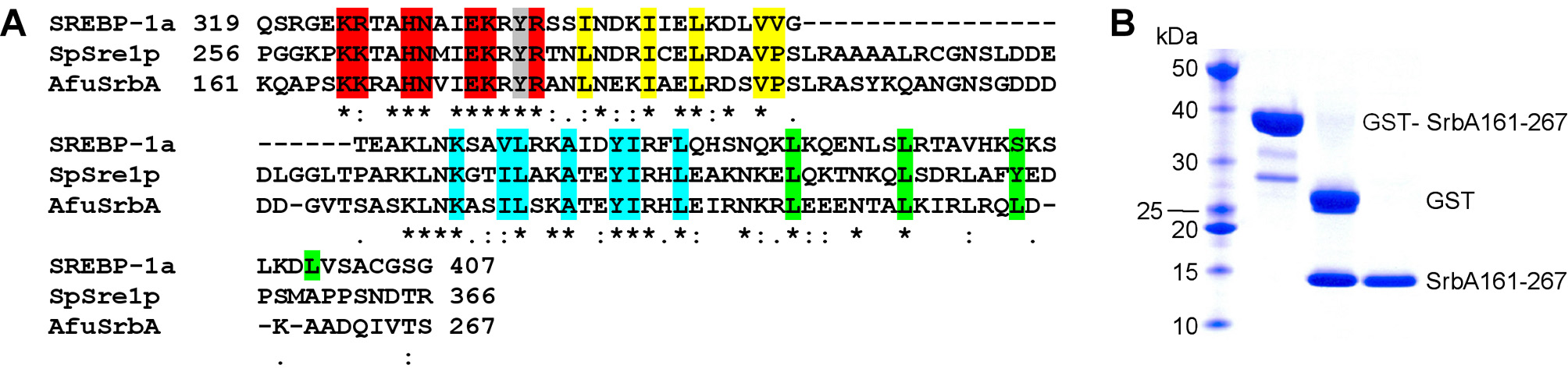


**Figure 1.** **(A)** Partial sequence alignment of the bHLHZ DNA-binding domains of human SREBP-1a, *S. pombe* Sre1 with *A. fumigatus* SrbA. Conserved residues are color-coded as follows: basic region (red), helix 1 (yellow), helix 2 (blue), and leucine zipper (green). The unique tyrosine residue of the SREBPs is marked in grey. (B) SDS-PAGE analysis of purified SrbA161-267.

DNA duplexes containing putative SREs from *srbA*, *hapX* and *hemA* promoter regions (Table 1) were tested for direct binding by SPR kinetic analysis. Injection of SrbA161-267 across the flow cell with a bound sequence element from the region of the *srbA* promoter (-735 to -726) with no mismatch compared to the Sre1p consensus sequence resulted in high affinity DNA binding responses that fit to a dissociation constant (K_D_) value of 0.63 nM (Fig. 2A). In contrast, only low-affinity binding (K_D_ > 300 nM) was observed for a DNA duplex mutant that served as negative control and altered the *srbA* SRE sequence from ATCATACGAT to ATATAACATA (Fig. 2B). Furthermore, high affinity SrbA161-267 binding was observed as well with putative SREs that had only one consensus mismatch. Kinetic binding responses on duplexes encoding *hapX* (-1340 to -1331) and *hemA* (-527 to -518) promoter regions fit with K_D_ values of 4.6 nM (Fig. 2D) and 4.2 nM (Fig. 2E), respectively. These data revealed a remarkable sequence homology between *S. pombe* and *A. fumigatus* SREs and will assist the identification of SrbA target genes and the refinement of SREs in *A. fumigatus*.

**
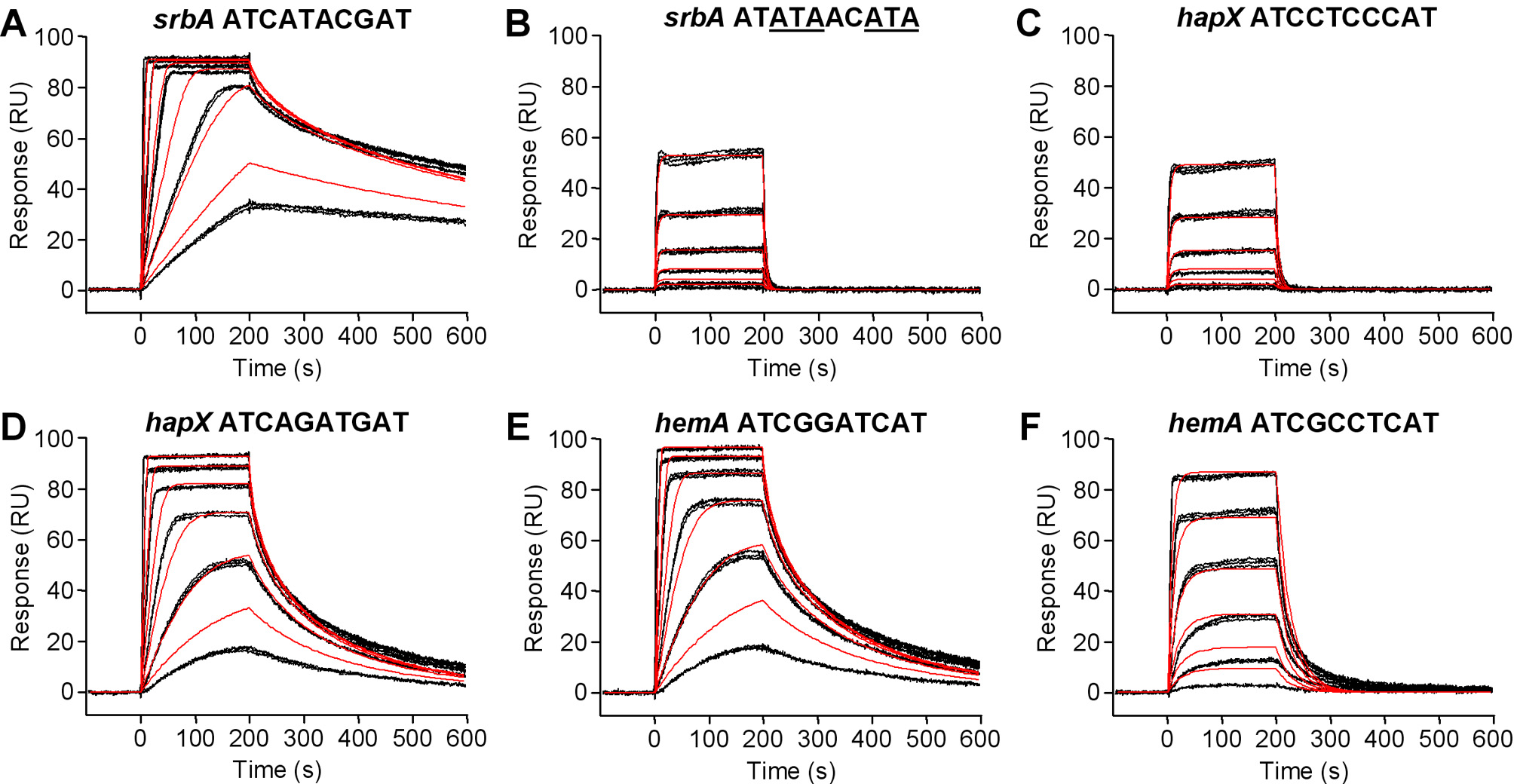
**

**Figure 2.** Real-time *in vitro* SPR binding analysis of SrbA to DNA encoding putative binding sites from *A. fumigatus* *srbA* **(A, B)**, *hapX* **(C, D)** and *hemA* **(E, F)** promoter regions. Sensorgrams of 100, 50, 25, 12.5, 6.25, and 3.13 nM SrbA161-267 binding injected in triplicate (black lines) are shown overlaid with the best fit derived from a 1:1 interaction model including a mass transport term (red lines).

# Table 1. Binding constants for putative SrbA binding sites

| **Gene** | **Positions** | **Strand** | **Sequence** | **k_a_ (M^-1^ s^-1^)** | **k_d_ (s^-1^)** | **K_D_ (nM)** |
| --- | --- | --- | --- | --- | --- | --- |
| *srbA* | -735 to -726 | Sense | ATCATACGAT | 1.48 ± 0.05 x 10^7^ | 9.27 ± 0.29 x 10^-3^ | 0.63 ± 0.04 |
| *srbA** | -735 to -726 | Sense | ATATAACATA | 5.92 ± 0.08 x 10^5^ | 2.29 ± 0.01 x 10^-1^ | 386 ± 7.3 |
| *hapX* | -774 to -783 | Antisense | ATCCTCCCAT | 5.68 ± 0.07 x 10^5^ | 1.61 ± 0.01 x 10^-1^ | 282 ± 5.1 |
| *hapX* | -1340 to -1331 | Sense | ATCAGATGAT | 3.10 ± 0.01 x 10^7^ | 1.44 ± 0.01 x 10^-1^ | 4.64 ± 0.03 |
| *hemA* | -527 to -518 | Sense | ATCGGATCAT | 1.41 ± 0.07 x 10^7^ | 5.88 ± 0.28 x 10^-2^ | 4.18 ± 0.41 |
| *hemA* | -338 to -347 | Antisense | ATCGCCTCAT | 1.03 ± 0.01 x 10^6^ | 3.66 ± 0.02 x 10^-2^ | 35.7 ± 0.52 |

* Mutated nucleotides are underlined

**Table 2. Oligonucleotides used in this study**

| Oligonucleotide | **Sequence (5‘→3‘)*** |
| --- | --- |
| BsrbApmm0 | Biotin-TTCCCGCGATCATACGATGTGCTCCG |
| srbApmm0 | CGGAGCACATCGTATGATCGCGGGAA |
| BsrbApmm0mut | Biotin-TTCCCGCGATATAACATAGTGCTCCG |
| srbApmm0mut | CGGAGCACTATGTTATATCGCGGGAA |
| BhapXpmm11 | Biotin-GCCTTTGAATGGGAGGATTCTTTATT |
| hapXpmm11 | AATAAAGAATCCTCCCATTCAAAGGC |
| BhapXpmm12 | Biotin-CAGATAAGATCAGATGATGAAGGGAT |
| hapXpmm12 | ATCCCTTCATCATCTGATCTTATCTG |
| BhemApmm11 | Biotin-TCCCCAGTATCGGATCATACGGCTAC |
| hemApmm11 | GTAGCCGTATGATCCGATACTGGGGA |
| BhemApmm12 | Biotin-CTGAGGCAATGAGGCGATGAGGCAAC |
| hemApmm12 | GTTGCCTCATCGCCTCATTGCCTCAG |

* Putative SrbA binding sites are underlined
